# Leaf-branch-root trait relationships in *Quercus rehderiana* across rocky and non-rocky desertification habitats in China

**DOI:** 10.7717/peerj.20367

**Published:** 2025-11-21

**Authors:** Xiaolong Bai, Shun Zou, Tu Feng, Dongpeng Lv, Bin He, Wangjun Li

**Affiliations:** 1 College of Ecological Engineering, Guizhou University of Engineering Science, Bijie, China; 2 Key Laboratory of Ecological Microbial Remediation Technology of Yunnan Higher Education Institutes, Dali University, Dali, China

**Keywords:** *Quercus rehderiana*, Trade-off, Plant functional traits, Rock desertification, Resource strategies

## Abstract

**Background:**

Plant leaves, branches, and roots synergistically govern survival, growth, and reproduction. However, while interspecific and community-level studies have advanced our understanding of organ coordination, intraspecific trait covariation remains poorly understood due to limited evidence.

**Methodology:**

This study investigated 28 functional traits across leaves, branches, and roots of *Quercus rehderiana*, a dominant species in rocky and non-rocky desertification forests, to evaluate intraspecific organ relationships. The traits, covering morphological, anatomical, and physiological aspects, reflect resource acquisition and utilization strategies. Standardized protocols were followed, with three replicates per individual for reliability.

**Results:**

Our results revealed no significant correlations among leaf, branch, and root traits in either forest type. Principal component analysis (PCA) of leaf traits indicated that the first axis was positively associated with water storage and utilization strategies, showing positive correlations with leaf thickness (LT), palisade mesophyll thickness (PT), and spongy mesophyll thickness (ST). The second axis exhibited a positive relationship with leaf nitrogen concentration (LNC) and leaf phosphorus concentration (LPC). For branch traits, the first axis reflected water transport efficiency, demonstrating positive associations with theoretical hydraulic conductivity (*K*_t_) and vessel density (VD). The second axis was positively correlated with branch N concentration (BNC) and branch phosphorus concentration (BPC). In root traits, the first axis aligned with root defense traits (positive correlation) but was inversely related to resource acquisition efficiency. The second axis showed a positive correlation with root N concentration (RNC) and root phosphorus concentration (RPC).

**Conclusions:**

Organ-specific trait decoupling in *Quercus rehderiana* reveals independent above- and belowground adaptations to water and nutrient limitations, challenging whole-plant economic spectrum assumptions. While consistent in rocky desertification forests, they differ from other ecosystems, highlighting context-dependence. Future research should expand across environmental gradients to disentangle trait relationships. This work highlights multidimensional approaches in functional ecology for understanding plant adaptation.

## Introduction

Plant functional traits encompass morphological, physiological, and anatomical characteristics that govern plant growth, reproduction, and survival (Violle et al., 2007). These traits reflect plant adaptations to environmental conditions and serve as indicators of ecological strategies employed by species to adapt to their habitats (Reich, 2014; Martini et al., 2021). As the primary photosynthetic organs, leaves are specialized for light interception and CO_2_ assimilation. Specific leaf area (SLA), defined as light-capturing surface area per unit dry mass, is a key indicator of carbon acquisition strategies (Lambers, Chapin & Pons, 1998; Wright et al., 2004). Leaf tissue thickness is a critical functional trait influencing multiple physiological processes, including resource-use efficiency, water retention, and photosynthetic performance (Garnier, Navas & Grigulis, 2016; Onoda et al., 2017). Stems provide structural support while facilitating water and nutrient transport, with wood density (WD) reflecting mechanical strength and pathogen resistance (Perez-Harguindeguy et al., 2016). Xylem traits such as VD, vessel diameter (*D*_v_), and hydraulic conductivity influence water transport efficiency and safety (Chave et al., 2009; Hacke et al., 2017; De Freitas, Da Cunha & Vitória, 2024). Roots absorb and transport soil nutrients, often doubling as storage organs (Fortunel, Fine & Baraloto, 2012; McCormack et al., 2015). Their architectural traits—including diameter, volume, length, area, and tissue density—collectively determine resource acquisition efficiency and ecological adaptation capacity (Hodge, 2009; Prieto et al., 2015). Dry matter content across organs (leaves, stems, and roots) reflect tissue longevity and growth rate (Wright & Cannon, 2001; Cornelissen et al., 2003; Díaz et al., 2016). Carbon, nitrogen, and phosphorus are fundamental elements for plant growth and reproduction. There is substantial information about the role of these three elements in plant physiological mechanisms, such as phosphorus in metabolic respiration, and nitrogen in photosynthetic pigments (McGroddy, Daufresne & Hedin, 2004; Onoda, Hikosaka & Hirose, 2004; Reich et al., 2008; Chen, Du & Liu, 2020; Bhatla & Lal, 2023; Du et al., 2023).

Plant ecological strategies are characterized by coordinated functional traits that reflect a fundamental trade-off between resource acquisition and conservation adaptations, collectively forming an economic spectrum (Reich, 2014; Díaz et al., 2016; Carmona et al., 2021). Over recent decades, studies of plant economic spectrum have primarily focused on interspecific trait variation. In contrast, intraspecific trait variability and its relationship with economic spectrum remain poorly understood (Garnier et al., 2001; Kraft, Godoy & Levine, 2015; Da et al., 2025). This gap is particularly noteworthy given that biotic and abiotic filters operate at the individual level before manifesting at the species level (Violle et al., 2012; Siefert et al., 2015; Blouin, Dubs & Ponge, 2024). Consequently, neglecting intraspecific variation may lead to biased ecological interpretations (Chacón-Labella et al., 2023; Palacio et al., 2025). Additionally, methodological challenges in fine root sampling and trait measurements have resulted in comparatively limited research on belowground traits relative to aboveground (leaf and stem) traits (Freschet et al., 2010). These limitations have fueled ongoing debate about whether aboveground and belowground traits exhibit coordinated or decoupled patterns of variation (Weemstra et al., 2021; Liu et al., 2023; Da et al., 2025).

Two competing hypotheses explain leaf-stem relationships: the life history theory proposes trait coordination (Grime et al., 1997; De la Riva et al., 2016; Wang et al., 2017; Kawai & Okada, 2019), while economic spectra hypothesis suggests their independence (Wright et al., 2007; Baraloto et al., 2010; Fortunel, Fine & Baraloto, 2012; Weemstra et al., 2016). The trait coordination hypothesis argues that plants in resource-limited environments optimize traits for resource acquisition and conservation (Grime et al., 1997; De la Riva et al., 2016). For instance, slow-growing species often develop thick, dense leaves (high leaf mass per area (LMA)) and dense stems (high WD) to improve durability and stress resistance (Wright et al., 2007; Kawai & Okada, 2019). Some plants may also combine high leaf mass per area with high WD to minimize cavitation risk while conserving water (Méndez-Alonzo et al., 2012; De la Riva et al., 2016). In contrast, the trait decoupling hypothesis posits that leaves and stems evolve under different selective pressures: leaves prioritize photosynthetic efficiency (Wright et al., 2007), while stems focus on hydraulic conductivity and mechanical support (Baraloto et al., 2010; Fortunel, Fine & Baraloto, 2012). In resource-rich environments, where strict trait coordination is unnecessary, decoupling may occur (Freschet et al., 2010; Weemstra et al., 2016). For example, fast-growing species can pair low-LMA leaves (for rapid light capture) with low-density stems (for efficient transport) without sacrificing survival (Ishida et al., 2008).

The coordination between stem and leaf traits is mediated by a continuous vascular system of xylem and phloem tissues (Pratt et al., 2007), which facilitates resource transport and signal transduction. This vascular connectivity explains the strong correlations observed in both structural (*e.g.*, WD) and chemical traits (Freschet et al., 2010; De la Riva et al., 2016; Wang et al., 2017). However, environmental stressors like water availability can modulate these relationships. Drought conditions, for instance, may alter hydraulic conductivity and shift carbon allocation, leading to divergent adjustments in stem and root traits (De la Riva et al., 2016). In extreme cases—such as prolonged water limitation or nutrient deprivation—complete decoupling of stem-root trait coordination can occur (Baraloto et al., 2010; Fortunel, Fine & Baraloto, 2012). Such decoupling events underscore the plasticity of plant vascular systems under abiotic stress. Resources may be preferentially allocated to stems (*e.g.*, to maintain hydraulic safety) or roots (*e.g.*, to enhance foraging efficiency), thereby disrupting typically conserved trait relationships.

Similarly, studies on leaf-root relationships present divergent hypotheses—ranging from coordinated functional traits (Kerkhoff et al., 2006; Freschet et al., 2010; Reich, 2014; De la Riva et al., 2016) or independence (Craine & Lee, 2003; Craine et al., 2005)—with environmental factors such as water availability, soil nutrients, and temperature further modulating these patterns (Fort, Jouany & Cruz, 2013; Geng et al., 2014; De la Riva et al., 2016). Coordinated leaf-root traits reflect a balanced carbon-nitrogen economy, where plants optimize photosynthesis (*via* leaf traits) and resource acquisition (*via* root traits) under stable conditions (Reich, 2014; De la Riva et al., 2016). However, decoupling may occur under specific stressors: nutrient limitation favors root proliferation at the expense of leaf growth (Craine & Lee, 2003; Craine et al., 2005), while water stress induces root elongation (*e.g.*, deep rooting) coupled with reduced leaf area (LA) (Cordlandwehr et al., 2013; Fort, Jouany & Cruz, 2013). Low soil nutrient availability may also increase specific root length (SRL) without corresponding leaf trait adjustments (Freschet et al., 2013), whereas extreme temperatures can impair phloem transport, disrupting source–sink coordination (Geng et al., 2014). In tropical forests, trait independence may emerge from competitive pressures—light competition drives canopy leaf optimization (*e.g.*, thicker leaves), belowground competition diversifies root strategies (Fortunel, Fine & Baraloto, 2012), and hydraulic segmentation allows stems/roots to endure drought while safeguarding leaves (Baraloto et al., 2010; Choat et al., 2018). Therefore, it remains unclear whether the leaves, stems, and roots of the same plant exhibit decoupling or coupling under different environmental stresses. Thus, elucidating intraspecific trait-trait relationships is critical to unravel plant adaptive strategies (Zhou, Cieraad & Van Bodegom, 2022).

The relationships among traits across different plant organs may also vary due to divergent environmental conditions (Cordlandwehr et al., 2013) or distinct evolutionary constraints acting on different plant communities (Heberling & Fridley, 2012). Consequently, further investigations are required to examine the coordination patterns among leaf, stem, and root traits across varying environmental conditions. Such comprehensive studies will enable us to draw more conclusions regarding the existence and nature of plant economic spectra at both species and community levels (Zhou, Cieraad & Van Bodegom, 2022). *Quercus rehderiana*, a species of the genus *Quercus* in the Fagaceae family, has important economic and ecological value (Lu et al., 2018). It is widely distributed in both rocky desertification and non-rocky desertification environments (Wang et al., 2023), offering a rare opportunity to explore the intraspecific variation in leaf, stem and root functional traits across different environments. In this study, we measured 28 functional traits of *Quercus rehderiana* from leaves, branches, and fine roots (diameter ≤ two mm) in rocky and non-rocky desertification forests, including key morphological, anatomical, and chemical traits (Table 1). The primary objective was to investigate whether functional traits across organs (leaves, branches, roots) within the same species maintain consistent correlations or exhibit decoupling under varying environmental stressors. We hypothesize that trait covariation occurs among leaves, branches, and roots of *Quercus rehderiana* in harsh karst rocky desertification forest environments, whereas these traits become decoupled in non-rocky desertification conditions.

**Table 1:** The 28 measured traits of plant organ (group).

Trait	Abbreviation	Unit	Group
Leaf area	LA	cm^2^	Leaf
Leaf thickness	LT	um	Leaf
Specific leaf area	SLA	cm^2^ g^−1^	Leaf
Leaf dry matter content	LDMC	g g^−1^	Leaf
Adaxial epidermis thickness	Ada	um	Leaf
Abaxial epidermis thickness	Aba	um	Leaf
Palisade mesophyll thickness	PT	um	Leaf
Spongy mesophyll thickness	ST	um	Leaf
Leaf carbon concentration	LCC	mg g^−1^	Leaf
Leaf nitrogen concentration	LNC	mg g^−1^	Leaf
Leaf phosphorus concentration	LPC	mg g^−1^	Leaf
Wood density	WD	g cm^−3^	Branch
Vessel diameter	*D*_V_	um	Branch
Mean biggest vessel diameter	*D*_max_	um	Branch
Vessel density	VD	no. mm^2^	Branch
Theoretical hydraulic conductivity	*K*_t_	kg m^−1^ s^−1^ MPa^−1^	Branch
Branch carbon concentration	BCC	mg g^−1^	Branch
Branch nitrogen concentration	BNC	mg g^−1^	Branch
Branch phosphorus concentration	BPC	mg g^−1^	Branch
Root diameter	RD	mm	Root
Root volume	RV	cm^3^	Root
Specific root length	SRL	cm g^−1^	Root
Specific root area	SRA	cm^2^ g^−1^	Root
Root dry matter content	RDMC	g g^−1^	Root
Root tissue density	RTD	g cm^−3^	Root
Root carbon concentration	RCC	mg g^−1^	Root
Root nitrogen concentration	RNC	mg g^−1^	Root
Root phosphorus concentration	RPC	mg g^−1^	Root

## Materials & Methods

### Study area

This research was conducted in Weining County, Bijie City, located in the northwest of Guizhou Province in southwestern China (103°36′–104°30′E, 26°30′ − 27°25′N). The study area lies within the mid-subtropical zone and is influenced by a monsoon climate, with an average annual precipitation of approximately 1,000 mm and a mean annual temperature of 12 °C. Characterized by its low latitude, high elevation, and plateau mountainous terrain, the region has an average altitude of 2,200 m. The predominant soil type is limestone soil, with a pH of 5.50. Due to grazing and agricultural activities, the vegetation in the herbaceous and shrub layers is sparse. The shrub layer is primarily composed of *Rhododendron simsii*, *Cotoneaster franchetii*, and *Corylus yunnanensis*, while the herbaceous layer includes species such as *Rubia cordifolia*, *Viola philippica*, and *Plantago asiatica* (He et al., 2021).

### Experimental design

**Figure 1:**
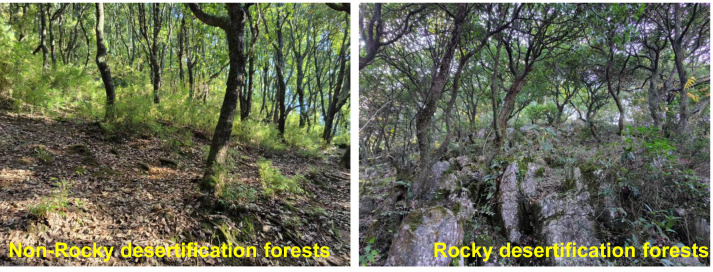
Features of rocky desertification and non-rocky desertification plots.

The classification of rocky and non-rocky desertification in this study was based on vegetation coverage, rock exposure rate, and average soil thickness (Li et al., 2004). Rocky desertification was defined as having low vegetation coverage (<35%), high rock exposure rate (>60%), and thin soil layer (<15 cm), whereas non-rocky desertification exhibited higher vegetation coverage (>35%), lower rock exposure rate (<60%), and thicker soil layer (>15 cm). From July to September 2021, five 20 × 20 m quadrats were established in both rocky desertification and non-rocky desertification forests (Fig. 1). The preliminary survey indicated that five sample plots per type were sufficient to represent the dominant species and their distribution patterns. Due to high spatial heterogeneity in rocky desertification areas, these plots also captured microhabitat variations (*e.g.*, soil depth, rock exposure) while ensuring sampling feasibility. Each quadrat was further divided into four 10 × 10 m subplots to facilitate subsequent plant tissue sampling.

### Sampling

Within each of three 10 × 10 m quadrats, one mature *Quercus rehderiana* individual with a diameter at breast height (DBH) of 20–30 cm was selected as a sample from quadrat center. From the canopy of each tree, three branches were collected, including healthy sun-exposed mature leaves and three stem segments (approximately one cm in diameter and 10 cm in length). Fine roots (diameter ≤ two mm) were excavated 50 cm from the trunk in the four cardinal directions (east, west, south, and north) around the main root, with three fine roots collected per direction. In each 20× 20 m quadrat, a total of 9 branch samples and 36 fine root samples were obtained from three trees. Across the rocky desertification and non-rocky desertification quadrats, 135 stems samples and 540 fine root samples were collected from 15 individuals. All samples were stored in sealed plastic bags with moistened paper towels.

### Trait measurements

Leaf functional traits (Table 1) were measured following the methodologies outlined by Cornelissen et al. (2003) and Perez-Harguindeguy et al. (2016). For each individual, five leaf samples were collected. Leaf area (LA, cm^2^) was measured using an HP Scanjet M231 scanner and ImageJ software (URL: https://imagej.en.softonic.com/ accessed on 22 May 2023). Leaf fresh weight was determined using an electronic balance (precision: 0.0001 g), followed by oven-drying at 70 °C for 48 h. Specific leaf area (SLA, cm^2^ g^−1^) and leaf dry matter content (LDMC, g g^−1^) were calculated as the ratio of LA to dry weight and dry weight to fresh weight, respectively. Cross-sectional images of 5–7 leaf tissues were captured using a Leica DM2500 Binocular Biological Microscope (Wetzlar, Germany). Leaf thickness (LT, µm), adaxial epidermis thickness (Ada, µm), abaxial epidermis thickness (Aba, µm), palisade mesophyll thickness (PT) (µm), and spongy mesophyll thickness (ST) (µm) were measured in ImageJ (accessed on 22 May 2023, available at: https://imagej.en.softonic.com/). Five replicate measurements were taken from each tissue parameter per image.

For branch traits, nine *Quercus rehderiana* individuals with branch diameters of approximately one cm were selected both rocky and non-rocky desertification forests (sample size reduced due to measurement losses). Each individual was measured three times. After removing bark and pith, wood volume was determined *via* water displacement. Samples were then oven-dried at 80 °C for 72 h, and WD (g cm^−3^) was calculated as dry weight divided by volume. One end of each segment was trimmed with a razor blade to ensure a perpendicular cross-section. Images were captured using an Ultra-depth Digital Microscope (Yiweishike Technology Co., Ltd., Chengdu, China), with five-eight images per individual. Vessel major and minor axes were measured in ImageJ (accessed on 22 May 2023, available at: https://imagej.en.softonic.com/), and VD (no mm^−2^) was calculated. Vessel diameter (*D*_V_, µm) followed Lewis (1992):

\begin{eqnarray*}{D}_{V}={ \left[ 32(ab)^{3}/({a}^{2}+{b}^{2}) \right] }^{1/4} \end{eqnarray*}

where *a* and *b* are the major and minor axis radii, respectively. The theoretical hydraulic conductivity (*K*_*t*_, kg m^−1^ s^−1^ MPa^−1^) was calculated as (Tyree & Ewers, 1991):

\begin{eqnarray*}{K}_{t}=(\pi \rho )/128\eta A \left[ \sum _{i}^{n} \left( {D}_{Vi}^{4} \right) \right] \end{eqnarray*}

where *ρ* is water density (997.05 kg m^−3^ at 25 °C), *η* is water viscosity (0.89 ×10^−9^ MPa s^−1^), *A* is the microscope field area, and *n* is the total vessel count. The 15 largest vessels per image were measured to determine the average maximum diameter (*D*_*m*__*a*__*x*_, µm), as they correlate with water transport efficiency (Zhang et al., 2019).

Roots were rinsed with tap water, washed three times with distilled water, and blotted dry. Fresh weight was recorded (precision: 0.0001 g). Roots were scanned at 1,200 dpi using a Microtek scanMaker i850 (Delhi, India), oven-dried at 65 °C for 72 h, and weighed. Morphological traits-root length (RL, cm), root diameter (RD, mm), root volume (RV, cm^3^), and surface area (SA, cm^2^)—were analyzed using DJ-GX02 root analysis software (Dianjiang Technology Co., Ltd., Shanghai, China). Derived traits included specific root length (SRL, cm g^−1^; root length divided by dry weight), specific root area (SRA, cm^2^ g^−1^; root surface area divided by dry weight), root tissue density (RTD, g cm^−3^; dry weight divided by root volume), and root dry matter content (RDMC, g g^−1^; dry weight divided by fresh weight) were calculated.

Dried leaf, branch, and fine root samples were ground into powder to determine the total carbon, nitrogen, and phosphorus concentrations. Total carbon and nitrogen concentrations (mg g^−1^) were measured using a Dumas-type combustion C-N elemental analyzer (Vario MAX CN, Elementar Analysensysteme GmbH, Hanau, Germany). Total phosphorus concentration (mg g^−1^) was determined with an inductively coupled plasma atomic-emission spectrometer (iCAP 7400, Thermo Fisher Scientific, Bremen, Germany).

### Statistical analysis

Data analysis was performed using the mean values of each individual plant. Prior to analysis, the data were log_10_-transformed to improve normality. To determine whether a consistent economic arrangement exists among the organs of karst rocky and non-rocky desertification forests, as well as how different organs coordinate with each other, we employed principal component analysis (PCA) to examine trait correlations across organs. This approach allowed us to assess whether functional differences were concentrated along a single dimension (with similar loadings across organs) or whether distinct organs or trait combination explained independent and significant portions of the overall functional inertia (Vleminckx et al., 2021). We then used PCA loading axis scores as proxies for plant organ economics. By fitting species scores from the first PCA axis for pairs of organs using general linear regression models, we evaluated the association between the trait spectra of each organ and those of other organs (Freschet et al., 2010). All statistical analyses and visualizations were conducted in R 4.4.0 using the packages *ape*, *FactoMiner*, *smatr*, *vegan*, *ggplot2* and *stats*.

## Results

Analysis of plant trait covariation reveals distinct correlation patterns at the organ level. Intra-organ functional traits (within leaf, stem, or root systems) consistently exhibit strong interdependencies, whereas inter-organ trait relationships showed significantly weaker associations (Tables S1, S2). In rocky desertification forests, leaf traits displayed the following relationships: LT was significantly positively correlated with LA (*R*^2^ = 0.55, *P* < 0.05) and leaf carbon concentration (LCC) (*R*^2^ = 0.64, *P* < 0.05), while SLA was negatively correlated with LDMC (*R*^2^ = −0.69, *P* < 0.01). In branches, VD was correlated positively with both WD (*R*^2^ = 0.67, *P* < 0.05) and *K*_t_ (*R*^2^ = 0.91, *P* < 0.01). For fine roots, RD showed a positive correlation with RV (*R*^2^ = 0.75, *P* <  0.01) but negative correlations with SRL (*R*^2^ = −0.87, *P* < 0.01) and SRA (*R*^2^ = −0.83, *P* < 0.01). Notably, SRL and SRA were strongly positively correlated (*R*^2^ = 0.99, *P* < 0.01) but both exhibited negative relationships with RTD (*R*^2^ = −0.53, *P* <  0.05; *R*^2^ = −0.64, *P* < 0.01) and RDMC (*R*^2^ = −0.62, *P* <  0.05; *R*^2^ = −0.66, *P* < 0.01). In non-rocky desertification forests, distinct patterns emerged: LT was positively correlated with PT (*R*^2^ = 0.71, *P* < 0.01) and ST (*R*^2^ = 0.97, *P* < 0.01). Leaf nitrogen concentration (LNC) was negatively associated with LDMC (*R*^2^ = −0.60, *P* < 0.05) but positively correlated with leaf phosphorus concentration (LPC) (*R*^2^ = 0.63, *P* < 0.05). Among branch traits, WD and *D*_V_ showed a positive relationship (*R*^2^ = 0.73, *P* < 0.05), as did VD and *K*_t_ (*R*^2^ = 0.98, *P* <  0.01). For fine roots, both RD (*R*^2^ = 0.74, *P* < 0.01) and RV (*R*^2^ = 0.77, *P* < 0.01) were positively associated with RDMC but inversely related to SRL (*R*^2^ = −0.80, *P* < 0.01; *R*^2^ = −0.71, *P* < 0.01) and SRA (*R*^2^ = −0.67, *P* < 0.01; *R*^2^ = −0.68, *P* < 0.01). SRA and SRL were strongly positively correlated (*R*^2^ = 0.94, *P* < 0.01), while both traits were negatively associated with RTD (*R* ^2^ = −0.73, *P* < 0.01). Additionally, root N concentration (RNC) and root phosphorus concentration (RPC) showed a negative correlation (*R*^2^ = −0.68, *P* < 0.01). Notably, the coordination patterns of inter-organ functional traits remained consistent across both rocky desertification and non-rocky desertification environments.

**Figure 2:**
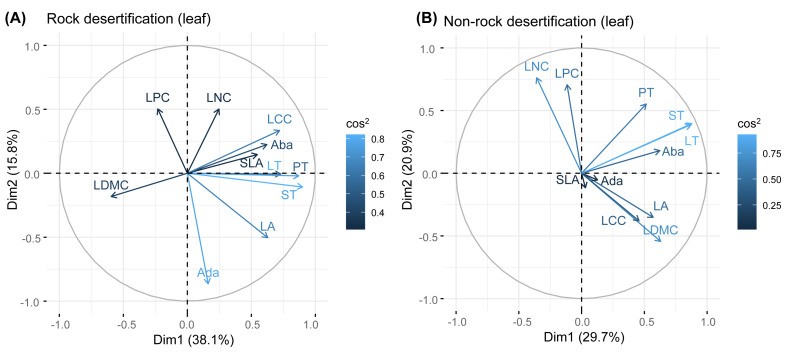
Principal components analysis of leaf traits. Abbreviations for traits are given in Table 1. LA, leaf area; LT, leaf thickness; SLA, specific leaf area; LDMC, leaf dry matter content; Ada, adaxial epidermis thickness; Aba, abaxial epidermis thickness; PT, palisade mesophyll thickness; ST, spongy mesophyll thickness; LNC, leaf nitrogen concentration; LCC, leaf carbon concentration; LPC, leaf phosphorus concentration.

**Figure 3:**
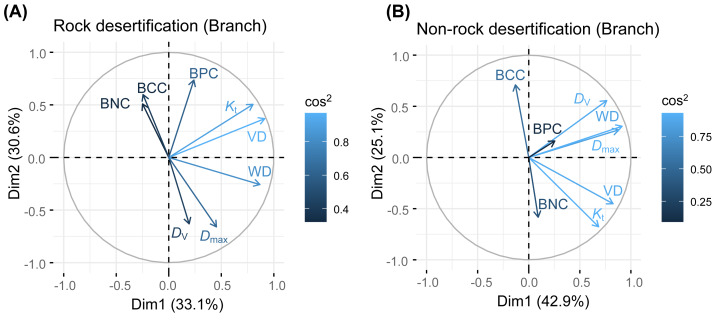
Principal components analysis of branch traits. Abbreviations for traits are given in Table 1. WD, wood density; VD, vessel density; *D*_*V*_, vessel diameter; *D*_max_, mean biggest vessel diameter; *K*_*t*_, theoretical hydraulic conductivity; BNC, branch nitrogen concentration; BCC, branch carbon concentration; BPC, branch phosphorus concentration.

**Figure 4:**
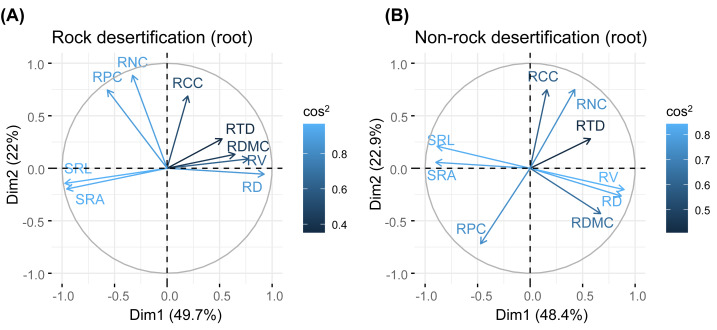
Principal components analysis of root traits. Abbreviations for traits are given in Table 1. RD, root diameter; RV, root volume; SRL, specific root length; SRA, specific root area; RDMC, root dry matter content; RTD, root tissue density; RNC, root nitrogen concentration; RCC, root carbon concentration; RPC, root phosphorus concentration.

Principal component analysis (PCA) of leaves, branches, and fine roots in rock and non-rock desertification forests revealed that the first axis explained varying proportions of the total variance (leaves: 38.1% *vs.* 29.7%; branches: 33.1% *vs.* 42.9%; roots: 49.7% *vs.* 48.4%, respectively; Figs. 2–4). In rocky desertification forests, the first PCA axis for leaves was primarily influenced by LCC, LT, PT, ST, and LA (Fig. 2A), whereas in non-rocky desertification forests, it was mainly driven by LT, ST, and LDMC (Fig. 2B). For branches, the first PCA axis in rocky desertification forests was predominantly associated with *K*_t_, VD and WD at the positive end (Fig. 3A), while in non-rock desertification forests, it was influenced by *K*_t_, VD, *D*_V_, *D*_max_ and WD at the positive end (Fig. 3B). Regarding roots, PCA in rock desertification forests captured 71.7% of the total variance across two principal components, with the first axis explaining 49.7% and the second axis 22.0% (Fig. 4A). In contrast, PCA in non-rock desertification forests accounted 71.3% of the total variance, with the first and second axes contribution 48.4% and 22.9%, respectively (Fig. 4B).

**Figure 5:**
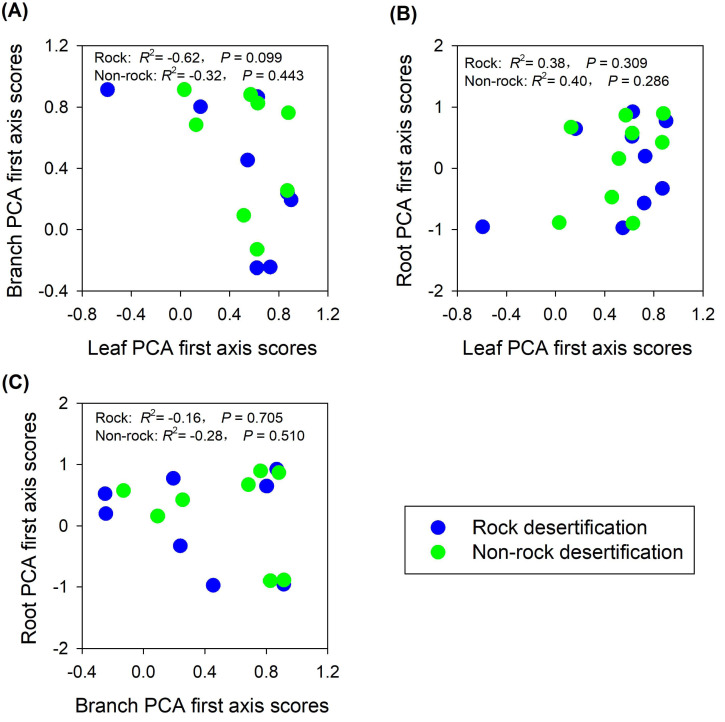
Correlations between economics spectra, as indicated by PCA first axis score, of different plant parts.

The findings partially supported our hypothesis, demonstrating trait decoupling among leaves, branches, and roots in both rocky and non-rocky desertification forests. PCA analysis revealed that: (1) first-axis scores of leaf traits showed nonsignificant negative correlations with branch traits in both forest types (rocky: *R*^2^ = −0.62, *P* = 0.099; non-rocky: *R*^2^ = −0.32, *P* = 0.443; Fig. 5A); (2) leaf and root traits exhibited nonsignificant positive relationships (rocky: *R*^2^ = 0.38, *P* = 0.309; non-rocky: *R* ^2^ = 0.40, *P* = 0.286; Fig. 5B); (3) Stem and root traits displayed nonsignificant negative correlations (rocky: *R*^2^ = −0.16, *P* = 0.705; non-rocky: *R*^2^ = −0.28, *P* = 0.510; Fig. 5C).

## Discussion

Understanding how plant organs coordinate remains a critical research priority, yet these interactions are still poorly understood and often inconsistent (Wright et al., 2006; De la Riva et al., 2016; Wang et al., 2017; Liu et al., 2023; Zhao et al., 2024). Functional trade-offs in resource allocation lead to coordinated trait strategies within each organ, which are best interpreted from a whole-plant perspective (Westoby et al., 2002; Baraloto et al., 2010; Silva et al., 2018; Luo et al., 2024). In this study, we examined correlations among 28 functional traits (leaf, branch, and root) of *Quercus rehderiana* in both rocky desertification and non-rocky desertification forests. Our results reveal a decoupled functional strategy system among the leaf, branch, and fine root traits of *Quercus rehderiana*, with organ-specific functional dimensions aligning orthogonally along distinct resource acquisition gradients (water, nutrient and light resources).

Our analysis revealed no significant association between the economic traits of leaves and branches in *Quercus rehderiana* across both forest types, suggesting that leaf and stem resources allocation strategies operate independent in both rocky and non-rocky habitats. This pattern of trait decoupling aligns with findings reported for tropical and subtropical tree species (Baraloto et al., 2010; Wang et al., 2017; Vleminckx et al., 2021), potentially reflecting species-specific optimizing of growth through differential resources partitioning among leaves, stems, and roots. However, contrasting evidence of coordinated leaf-stem trait spectra has been documented in subtropical Pacific islands ecosystems (Ishida et al., 2008) and certain forest biomes, including Mediterranean and Neotropical forests (De la Riva et al., 2016; Fortunel, Fine & Baraloto, 2012). These divergent results underscore the absence of a universal paradigm governing leaf-stem trait relationships across different ecosystems.

Principal component analysis (PCA) of leaf traits in both rocky desertification and non-desertification forests (Fig. 1) revealed a decoupled leaf economic spectrum, aligning with findings from tropical ecosystems (Wright et al., 2004; Baraloto et al., 2010; Fortunel, Fine & Baraloto, 2012). The first principal component (PC1) was positively associated with water storage traits (*e.g.*, LT, PT, and ST dimensions), reflecting water-use and storage strategies. In contrast, the second principal component (PC2) showed strong positive correlations with leaf N and P concentrations, representing nutrient acquisition strategies. This orthogonal relationship indicates that *Quercus rehderiana* leaves employ independent evolutionary strategies for water conservation and nutrient utilization.

The PCA results revealed a decoupling of economic gradients in branch traits (Fig. 2), a finding that contrasts with the conclusions of Chave et al. (2009) and Kawai & Okada (2019). Previous studies has demonstrated a fundamental trade-off between water transport efficiency (associated with low WD and fewer, larger vessels) and hydraulic safety (associated with high WD and more, smaller vessels) (Poorter et al., 2010; Zanne et al., 2010; Bittencourt, Pereira & Oliveira, 2016). In our study, the first PCA axis in both rocky and non-rocky desertification forests represents hydraulic strategies, showing positive correlation with VD, WD, and *K*_t_. Notably, *K*_t_ showed a significant positively correlated with VD but a non-significantly negative correlation with *D*_v_ across both rock and non-rock desertification forests (Table S1, S2). This pattern likely reflects an adaptive strategy of *Quercus rehderiana* to improve hydraulic efficiency through increased VD in arid environments. The second axis primarily captures nutrient allocation trade-offs, displaying positive correlations with branch nitrogen and phosphorus concentrations. The orthogonal relationship between these axes suggests independent evolutionary strategies for water transport and nutrient allocation in *Quercus rehderiana* branches.

Fine root traits showed no significant correlations between morphology and nutrient content (Table S1, S2). The PCA results indicated that the first axis reflects a trade-off in absorptive root size (length, surface area, volume), independent of trait organization and acquisition strategies (Fig. 3). Notably, the RTD of *Quercus rehderiana* fine roots in rock desertification forests covaried with other traits, including SRL, SRA, and RDMC. In contrast, fine root morphological traits displayed an orthogonal relationship with root nitrogen and phosphorus concentrations, implying evolutionary divergence in water/nutrient transport strategies. For non-rock desertification forests, RTD variation was independent of other traits, aligning with prior findings (Kramer-Walter et al., 2016; Vleminckx et al., 2021). This highlights the functional decoupling between root protective and acquisitive strategies.

Our findings align with observations reported in the Amazon region (Baraloto et al., 2010; Fortunel, Fine & Baraloto, 2012; Vleminckx et al., 2021), demonstrating that species invest independently in leaf, stem, and fine root tissues. Furthermore, our study reveals distinct nutrient and water utilization strategies across leaves, stems, and roots of *Quercus rehderiana* in both rocky and non-rocky desertification forest ecosystems. This functional differentiation among organs suggests compartmentalized resource allocation patterns, which may reflect divergent evolutionary histories or selective pressures—contrary to the assumption of a unified plant economics spectrum (Reich, 2014). Such organ-specific specialization could enhance environmental stress buffering, thereby contributing to ecosystem stability. From a conservation perspective, this adaptive capacity may bolster species resilience under escalating climate variability and anthropogenic pressures.

## Conclusions

Through a comparative analysis of leaf, branch, and fine root traits in *Quercus rehderiana* across rocky and non-rocky desertification forests, we demonstrate decoupled functional strategies among organs, with distinct variation patterns in above- and belowground traits. The orthogonal alignment of water-use strategies (first PCA axis) and nutrient-acquisition strategies (second PCA axis) in leaves, stems, and roots suggests that these traits evolve independently under contrasting environmental constraints—water scarcity *versus* nutrient limitation. By decoupling these processes, the study challenges traditional assumptions of tightly integrated whole-plant economics, revealing instead organ-specific adaptations to multidimensional resource gradients.

While our study reveals consistent trait patterns in *Quercus rehderiana* across habitats, observed divergence from other ecosystems (*e.g.*, tropical or Mediterranean forests) underscores the context-dependency of trait coordination. To generalize these findings, future research should expand to broader environmental gradients, geographical ranges, and congeneric species, employing meta-analyses to disentangle phylogenetic, biogeographic, and abiotic drivers of trait relationships. Our results provide a framework for understanding plant adaptive strategies in heterogeneous environments and emphasize the need for a multidimensional approach in functional ecology.

## Supplemental Information

10.7717/peerj.20367/supp-1 Supplemental Information 1 Data

10.7717/peerj.20367/supp-2 Supplemental Information 2 Supplemental tables

## Additional Information and Declarations

### Data Availability

The following information was supplied regarding data availability: The raw measurements are available in the Supplemental Files.
