# Supplementary material for: Leaf-branch-root trait relationships in Quercus rehderiana across rocky and non-rocky desertification habitats in China
Source: PeerJ. 2025 Nov 21;13:e20367. doi: 10.7717/peerj.20367 (PMC12642914; doi:10.7717/peerj.20367)
Supplement: Supplemental Information 2 [file peerj-13-20367-s002.docx]

**Table S1** Correlations among leaf, branch and root traits of *Quercus rehderiana* from rock desertification forests. Significant correlations are shown in bold.

| Trait | LT | SLA | LDMC | Ada | Aba | PT | ST | LCC | LNC | LPC | WD | *D*_V_ | | *D*_max_ | VD | | *K*_t_ | | BCC | | BNC | | BPC | | RD | | RV | | SRL | | SRA | | RDMC | | RTD | | RCC | | RNC | | RPC | |  |  |
| --- | --- | --- | --- | --- | --- | --- | --- | --- | --- | --- | --- | --- | --- | --- | --- | --- | --- | --- | --- | --- | --- | --- | --- | --- | --- | --- | --- | --- | --- | --- | --- | --- | --- | --- | --- | --- | --- | --- | --- | --- | --- | --- | --- | --- |
| LA | **0.55*** | 0.17 | -0.29 | 0.40 | 0.29 | 0.28 | **0.52*** | 0.45 | -0.18 | -0.31 | -0.35 | -0.38 | | -0.56 | -0.62 | | -0.37 | | 0.26 | | -0.18 | | -0.38 | | -0.44 | | -0.31 | | 0.24 | | 0.16 | | 0.13 | | -0.04 | | 0.01 | | **0.57*** | | **0.62*** | |  |  |
| LT |  | 0.25 | -0.28 | 0.21 | **0.52*** | **0.69**** | **0.89**** | **0.64*** | 0.25 | -0.29 | **-0.80*** | | -0.22 | -0.44 | | -0.65 | | -0.47 | | 0.47 | | -0.11 | | -0.27 | | 0.00 | | -0.13 | | -0.18 | | -0.22 | | **0.61*** | | 0.18 | | 0.10 | | 0.37 | | 0.19 | |  |
| SLA |  |  | **-0.69**** | 0.12 | 0.31 | 0.45 | 0.31 | 0.22 | 0.16 | -0.04 | -0.42 | -0.54 | | -0.51 | -0.23 | | -0.17 | | 0.14 | | **0.89**** | | 0.19 | | -0.37 | | -0.28 | | -0.02 | | -0.06 | | 0.18 | | 0.15 | | 0.01 | | -0.16 | | 0.20 | |  |  |
| LDMC |  |  |  | -0.03 | -0.25 | -0.37 | -0.31 | -0.48 | -0.11 | 0.06 | 0.09 | 0.47 | | 0.28 | 0.12 | | -0.03 | | -0.10 | | -0.62 | | 0.29 | | 0.34 | | 0.33 | | -0.16 | | -0.12 | | -0.16 | | 0.07 | | 0.13 | | 0.07 | | -0.13 | |  |  |
| Ada |  |  |  |  | -0.20 | 0.29 | 0.14 | -0.30 | -0.23 | -0.34 | -0.23 | 0.15 | | 0.30 | -0.56 | | -0.66 | | -0.25 | | -0.09 | | **-0.74*** | | | -0.08 | | 0.13 | | -0.17 | | -0.18 | | 0.24 | | 0.16 | | 0.26 | | 0.47 | | 0.47 | |  |
| Aba |  |  |  |  |  | 0.23 | 0.46 | 0.51 | 0.19 | -0.31 | -0.35 | -0.42 | | **-0.75*** | -0.32 | | -0.24 | | -0.02 | | -0.14 | | 0.12 | | -0.03 | | 0.10 | | -0.03 | | -0.05 | | 0.29 | | -0.08 | | 0.18 | | 0.06 | | 0.08 | |  |  |
| PT |  |  |  |  |  |  | **0.70**** | 0.34 | 0.22 | 0.13 | -0.60 | -0.52 | | -0.43 | -0.20 | | -0.05 | | 0.57 | | 0.29 | | -0.12 | | 0.08 | | -0.14 | | -0.31 | | -0.29 | | 0.41 | | -0.02 | | 0.06 | | -0.08 | | -0.19 | |  |  |
| ST |  |  |  |  |  |  |  | **0.57*** | 0.21 | -0.07 | **-0.75*** | | -0.28 | -0.50 | | -0.57 | | -0.34 | | 0.52 | | 0.01 | | -0.21 | | 0.02 | | -0.12 | | -0.25 | | -0.29 | | **0.53*** | | 0.21 | | 0.04 | | 0.25 | | 0.16 | |  |
| LCC |  |  |  |  |  |  |  |  | 0.13 | -0.01 | -0.47 | -0.08 | | -0.32 | -0.43 | | -0.06 | | 0.62 | | -0.06 | | -0.26 | | -0.34 | | **-0.54*** | | 0.34 | | 0.29 | | 0.30 | | -0.14 | | -0.30 | | 0.06 | | 0.12 | |  |  |
| LNC |  |  |  |  |  |  |  |  |  | -0.04 | -0.12 | -0.58 | | -0.32 | -0.09 | | -0.14 | | -0.19 | | 0.58 | | -0.22 | | 0.17 | | -0.05 | | -0.22 | | -0.24 | | 0.04 | | 0.11 | | 0.46 | | 0.23 | | -0.03 | |  |  |
| LPC |  |  |  |  |  |  |  |  |  |  | 0.31 | -0.41 | | -0.11 | **0.77*** | | **0.73*** | | 0.13 | | -0.05 | | 0.29 | | -0.02 | | -0.25 | | 0.08 | | 0.11 | | -0.10 | | -0.16 | | **-0.62*** | | **-0.56*** | | -0.47 | |  |  |
| WD |  |  |  |  |  |  |  |  |  |  |  | 0.08 | | 0.39 | **0.67*** | | 0.52 | | -0.56 | | -0.37 | | -0.10 | | 0.35 | | 0.20 | | 0.13 | | 0.20 | | -0.30 | | -0.39 | | 0.14 | | -0.02 | | -0.17 | |  |  |
| *D*_V_ |  |  |  |  |  |  |  |  |  |  |  |  | | **0.85**** | -0.09 | | -0.11 | | 0.01 | | -0.26 | | -0.13 | | 0.09 | | -0.02 | | 0.23 | | 0.25 | | 0.14 | | 0.33 | | -0.02 | | 0.34 | | 0.33 | |  |  |
| *D*_max_ |  |  |  |  |  |  |  |  |  |  |  |  | |  | 0.18 | | 0.09 | | -0.14 | | -0.21 | | -0.32 | | 0.24 | | 0.01 | | 0.14 | | 0.23 | | 0.00 | | 0.01 | | 0.16 | | 0.23 | | 0.07 | |  |  |
| VD |  |  |  |  |  |  |  |  |  |  |  |  | |  |  | | **0.91**** | | -0.07 | | -0.01 | | 0.48 | | 0.18 | | -0.26 | | 0.35 | | 0.47 | | -0.58 | | -0.48 | | -0.45 | | -0.52 | | -0.55 | |  |  |
| *K*_t_ |  |  |  |  |  |  |  |  |  |  |  |  | |  |  | |  | | 0.28 | | 0.08 | | 0.45 | | 0.02 | | -0.58 | | 0.51 | | 0.59 | | -0.60 | | -0.54 | | -0.50 | | -0.53 | | -0.47 | |  |  |
| BCC |  |  |  |  |  |  |  |  |  |  |  |  | |  |  | |  | |  | | 0.34 | | 0.26 | | -0.24 | | **-0.76*** | | 0.23 | | 0.21 | | -0.09 | | -0.06 | | -0.43 | | -0.30 | | -0.16 | |  |  |
| BNC |  |  |  |  |  |  |  |  |  |  |  |  | |  |  | |  | |  | |  | | 0.35 | | -0.67 | | -0.57 | | 0.22 | | 0.13 | | -0.49 | | 0.31 | | 0.14 | | -0.41 | | 0.10 | |  |  |
| BPC |  |  |  |  |  |  |  |  |  |  |  |  | |  |  | |  | |  | |  | |  | | -0.18 | | -0.32 | | 0.23 | | 0.19 | | -0.47 | | 0.30 | | -0.65 | | -0.63 | | -0.25 | |  |  |
| RD |  |  |  |  |  |  |  |  |  |  |  |  | |  |  | |  | |  | |  | |  | |  | | **0.78**** | | **-0.87**** | | **-0.83**** | | | 0.45 | | 0.30 | | 0.28 | | -0.29 | | **-0.66**** | | |
| RV |  |  |  |  |  |  |  |  |  |  |  |  | |  |  | |  | |  | |  | |  | |  | |  | | **-0.73**** | | **-0.67**** | | | 0.29 | | 0.17 | | 0.36 | | -0.15 | | -0.41 | |  |
| SRL |  |  |  |  |  |  |  |  |  |  |  |  | |  |  | |  | |  | |  | |  | |  | |  | |  | | **0.99**** | | | **-0.62*** | | **-0.53*** | | -0.32 | | 0.21 | | 0.45 | |  |
| SRA |  |  |  |  |  |  |  |  |  |  |  |  | |  |  | |  | |  | |  | |  | |  | |  | |  | |  | | **-0.66**** | | **-0.64**** | | -0.28 | | 0.17 | | 0.37 | |  |  |
| RDMC |  |  |  |  |  |  |  |  |  |  |  |  | |  |  | |  | |  | |  | |  | |  | |  | |  | |  | |  | | **0.56*** | | -0.12 | | -0.06 | | -0.15 | |  |  |
| RTD |  |  |  |  |  |  |  |  |  |  |  |  | |  |  | |  | |  | |  | |  | |  | |  | |  | |  | |  | |  | | -0.11 | | 0.00 | | 0.10 | |  |  |
| RCC |  |  |  |  |  |  |  |  |  |  |  |  | |  |  | |  | |  | |  | |  | |  | |  | |  | |  | |  | |  | |  | | 0.50 | | 0.22 | |  |  |
| RNC |  |  |  |  |  |  |  |  |  |  |  |  | |  |  | |  | |  | |  | |  | |  | |  | |  | |  | |  | |  | |  | |  | | **0.80**** | |  |  |

**Table S2** Correlations among leaf, branch and root traits of *Quercus rehderiana* from non-rock desertification forests. Significant correlations are shown in bold.

| Trait | LT | SLA | LDMC | Ada | Aba | PT | ST | LCC | LNC | LPC | WD | *D*_V_ | *D*max | VD | Kt | BCC | BNC | BPC | RD | RV | SRL | SRA | RDMC | RTD | RCC | RNC | RPC |
| --- | --- | --- | --- | --- | --- | --- | --- | --- | --- | --- | --- | --- | --- | --- | --- | --- | --- | --- | --- | --- | --- | --- | --- | --- | --- | --- | --- |
| LA | 0.34 | 0.40 | 0.42 | -0.31 | 0.30 | 0.09 | 0.26 | 0.36 | -0.31 | -0.23 | 0.23 | 0.13 | 0.38 | 0.64 | 0.60 | -0.45 | 0.13 | -0.47 | 0.04 | 0.18 | 0.34 | 0.34 | 0.33 | -0.19 | 0.19 | 0.01 | -0.22 |
| LT |  | -0.05 | 0.27 | 0.14 | 0.48 | **0.71**** | **0.97**** | 0.19 | -0.09 | 0.10 | 0.55 | 0.34 | 0.50 | 0.24 | 0.14 | 0.23 | -0.28 | -0.58 | 0.19 | 0.32 | 0.00 | 0.05 | 0.12 | -0.18 | 0.19 | 0.23 | **-0.53*** |
| SLA |  |  | -0.10 | -0.27 | 0.22 | -0.33 | 0.02 | 0.10 | 0.12 | 0.02 | 0.43 | 0.28 | 0.44 | **0.81**** | **0.74*** | -0.59 | 0.02 | 0.16 | -0.24 | -0.32 | 0.38 | 0.48 | 0.05 | -0.49 | -0.09 | -0.44 | 0.40 |
| LDMC |  |  |  | 0.23 | 0.44 | -0.10 | 0.29 | 0.46 | **-0.60*** | -0.24 | 0.20 | 0.57 | 0.47 | -0.04 | -0.32 | 0.40 | -0.04 | 0.17 | 0.08 | 0.23 | 0.27 | 0.23 | 0.31 | -0.07 | 0.16 | 0.42 | -0.20 |
| Ada |  |  |  |  | 0.07 | 0.13 | 0.14 | -0.41 | -0.40 | -0.18 | -0.10 | 0.41 | 0.20 | -0.57 | -0.53 | 0.20 | -0.29 | -0.08 | 0.10 | 0.07 | -0.11 | -0.09 | 0.02 | 0.04 | -0.25 | 0.41 | -0.34 |
| Aba |  |  |  |  |  | 0.07 | 0.51 | 0.23 | 0.04 | 0.33 | -0.02 | 0.15 | 0.10 | -0.02 | -0.20 | 0.24 | **-0.74*** | -0.46 | 0.29 | 0.24 | 0.03 | 0.17 | 0.08 | -0.37 | 0.30 | 0.22 | -0.23 |
| PT |  |  |  |  |  |  | **0.66**** | -0.01 | 0.18 | -0.04 | 0.21 | -0.07 | 0.08 | 0.01 | 0.01 | 0.40 | -0.11 | **-0.69*** | 0.14 | 0.24 | -0.24 | -0.27 | 0.01 | 0.19 | -0.10 | 0.14 | -0.36 |
| ST |  |  |  |  |  |  |  | 0.22 | -0.11 | 0.12 | 0.50 | 0.34 | 0.47 | 0.15 | 0.02 | 0.26 | -0.23 | -0.51 | 0.15 | 0.26 | 0.04 | 0.09 | 0.06 | -0.20 | 0.13 | 0.20 | -0.42 |
| LCC |  |  |  |  |  |  |  |  | -0.19 | -0.25 | 0.31 | 0.06 | 0.23 | 0.44 | 0.21 | 0.03 | 0.37 | 0.01 | 0.05 | 0.17 | 0.25 | 0.28 | 0.20 | -0.25 | -0.15 | -0.29 | 0.37 |
| LNC |  |  |  |  |  |  |  |  |  | **0.63*** | 0.10 | -0.23 | -0.27 | 0.03 | 0.01 | 0.36 | -0.49 | 0.03 | -0.31 | -0.35 | 0.02 | 0.04 | -0.41 | -0.08 | -0.01 | -0.46 | 0.30 |
| LPC |  |  |  |  |  |  |  |  |  |  | 0.07 | -0.12 | -0.24 | -0.31 | -0.37 | 0.27 | **-0.67*** | -0.02 | -0.43 | -0.41 | 0.32 | 0.27 | **-0.68**** | -0.07 | **0.52*** | 0.01 | -0.17 |
| WD |  |  |  |  |  |  |  |  |  |  |  | **0.73*** | **0.85**** | 0.61 | 0.42 | 0.16 | -0.04 | 0.19 | -0.39 | -0.24 | 0.46 | 0.55 | 0.02 | **-0.93**** | 0.00 | -0.22 | -0.01 |
| *D*_V_ |  |  |  |  |  |  |  |  |  |  |  |  | **0.93**** | 0.31 | 0.11 | 0.13 | -0.25 | 0.31 | -0.19 | 0.02 | 0.21 | 0.30 | 0.22 | **-0.68*** | -0.39 | 0.18 | -0.14 |
| *D*_max_ |  |  |  |  |  |  |  |  |  |  |  |  |  | 0.57 | 0.38 | 0.00 | -0.09 | 0.16 | -0.11 | 0.09 | 0.18 | 0.28 | 0.33 | **-0.78*** | -0.35 | 0.06 | -0.15 |
| VD |  |  |  |  |  |  |  |  |  |  |  |  |  |  | **0.93**** | -0.30 | 0.24 | 0.07 | 0.17 | 0.21 | -0.06 | 0.02 | 0.54 | -0.48 | 0.11 | -0.44 | 0.22 |
| *K*_t_ |  |  |  |  |  |  |  |  |  |  |  |  |  |  |  | -0.50 | 0.34 | 0.01 | 0.30 | 0.31 | -0.22 | -0.15 | 0.58 | -0.30 | 0.01 | -0.56 | 0.13 |
| BCC |  |  |  |  |  |  |  |  |  |  |  |  |  |  |  |  | -0.17 | 0.17 | -0.20 | -0.23 | 0.15 | 0.13 | -0.16 | -0.13 | 0.25 | 0.28 | 0.12 |
| BNC |  |  |  |  |  |  |  |  |  |  |  |  |  |  |  |  |  | 0.32 | 0.12 | 0.07 | 0.00 | -0.07 | 0.37 | 0.26 | -0.43 | -0.43 | 0.62 |
| BPC |  |  |  |  |  |  |  |  |  |  |  |  |  |  |  |  |  |  | -0.34 | -0.31 | 0.34 | 0.27 | 0.10 | 0.05 | -0.18 | -0.53 | **0.74*** |
| RD |  |  |  |  |  |  |  |  |  |  |  |  |  |  |  |  |  |  |  | **0.89**** | **-0.80**** | **-0.67**** | **0.74**** | 0.16 | 0.06 | 0.20 | -0.26 |
| RV |  |  |  |  |  |  |  |  |  |  |  |  |  |  |  |  |  |  |  |  | **-0.71**** | **-0.68**** | **0.77**** | 0.36 | 0.03 | 0.18 | -0.38 |
| SRL |  |  |  |  |  |  |  |  |  |  |  |  |  |  |  |  |  |  |  |  |  | **0.94**** | -0.50 | -0.46 | 0.07 | -0.24 | 0.20 |
| SRA |  |  |  |  |  |  |  |  |  |  |  |  |  |  |  |  |  |  |  |  |  |  | -0.44 | **-0.73**** | -0.01 | -0.31 | 0.28 |
| RDMC |  |  |  |  |  |  |  |  |  |  |  |  |  |  |  |  |  |  |  |  |  |  |  | 0.12 | -0.05 | 0.01 | -0.03 |
| RTD |  |  |  |  |  |  |  |  |  |  |  |  |  |  |  |  |  |  |  |  |  |  |  |  | 0.16 | 0.32 | -0.34 |
| RCC |  |  |  |  |  |  |  |  |  |  |  |  |  |  |  |  |  |  |  |  |  |  |  |  |  | 0.51 | -0.50 |
| RNC |  |  |  |  |  |  |  |  |  |  |  |  |  |  |  |  |  |  |  |  |  |  |  |  |  |  | **-0.68**** |
